# Easily Satisfied!

**Published:** 1898-09-10

**Authors:** 


### EASILY SATISFIED!

After careful consideration the metropolitan magistrates have decided upon a form of certificate for issue in cases where they are satisfied that an applicant has a conscientious objection to the vaccination of his child. The form is simple enough. So-and-so "has this day stated to me that he conscientiously believes that vaccination will be prejudicial to the health of the said child, and that he has a conscientious objection to the child being vaccinated on that ground; and I am satisfied that he has such conscientious belief." When, however, we inquire how the magistrates are satisfied as to a man's belief, we find that this difficulty is made simpler still. "They will not require applicants to be sworn; but, if satisfied upon a verbal statement that they have the conscientious objection, will give the certificate." Truly, they are easily satisfied; but as no doubt this was the very intention of those who passed the clause in question, we can raise no objection to their proceedings. Now, however, that we see the practical effect of this wonderful clause, one must rather smile at the fight which was raised over Sir Walter Foster's amendment. The fact is that in the House of Commons politics always preponderate over measures; and this must be taken as explaining much.

